# Sialolithiasis in the Left Submandibular Gland: A Case

**DOI:** 10.7759/cureus.48999

**Published:** 2023-11-18

**Authors:** Prasanna R Sonar, Aarati Panchbhai, Pooja Dhole

**Affiliations:** 1 Oral Medicine and Radiology, Sharad Pawar Dental College and Hospital, Datta Meghe Institute of Higher Education and Research, Wardha, IND; 2 Oral Medicine and Radiology, Vidarbha Youth Welfare Society Dental College and Hospital, Amravati, IND

**Keywords:** sialolith, sialolithiasis, sialolithiasis submandibular gland disease, submandibular gland, disorders of the salivary glands

## Abstract

The most common illness affecting the salivary glands is submandibular gland sialoliths. The size of the sialolith and the patient's clinical history mainly influence how this salivary system abnormality is treated. This diagnosis is suggested by a history of salivary gland pain or swelling, particularly during mastication. Palliative therapy combined with conservative therapies, such as the milking of the ducts, can effectively treat small and accessible stones. When a stone or stones are large and inaccessible, surgical therapy should be considered if conservative approaches have not proven to be effective. A case of sialolithiasis affecting the left submandibular salivary gland is described in this article. Under local anesthesia, sialolith was removed following the opening of the duct. The wound was closed with sutures, and the patient was advised to practice tongue exercises and to maintain good oral hygiene.

## Introduction

The prevalent benign condition sialolithiasis is defined by the accumulation of stones within the ducts of the salivary glands. About 1.2% of unilateral large salivary gland swellings are caused by sialolithiasis, which is thought to be the most prevalent salivary gland condition. With an 80% occurrence rate, the submandibular gland is most predisposed to sialolithiasis, followed by the parotid and sublingual glands. The typical age range for sialolithiasis cases is 30-60 years old. Just 3% of all cases of sialolithiasis have been documented in youngsters, making it uncommon in this age group. The impact on men is twice that on women [1-3]. This article describes a case of clinical left-sided mandibular sialolithiasis that was effectively treated with salivary gland excision from the submandibular region. The article's goal is to describe a case of sialolithiasis and overview its etiology, diagnostic modalities, and treatment options.

## Case presentation

A 40-year-female reported to Sharad Pawar Dental College and Hospital with a principal concern with swelling and intermittent pain during mastication on the floor's left side of the mouth for one month. There was no history of trauma, and the swelling had been gradually growing larger over the previous ten years. Previous dental and medical histories were irrelevant. Upon extraoral examination, the face showed bilateral symmetry. Temporomandibular joint (TMJ) moments were synchronous and smooth on both sides. Palpable regional lymph nodes were absent. There were no pulsations or bruits.

**Figure 1:**
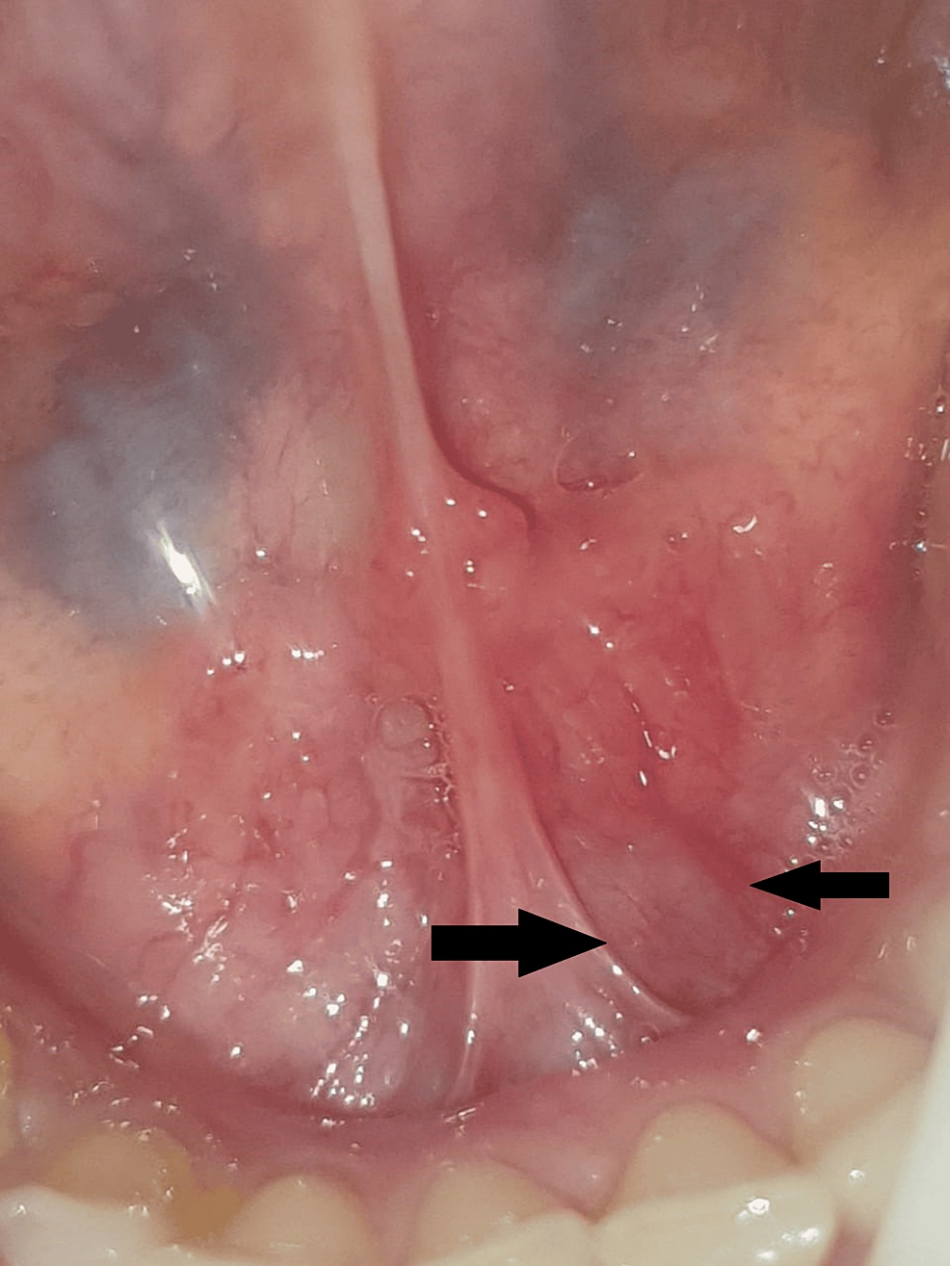
Intraoral photo showing diffuse swelling present on the left side of the floor of the mouth. Image Credit: Prasanna R. Sonar

**Figure 2:**
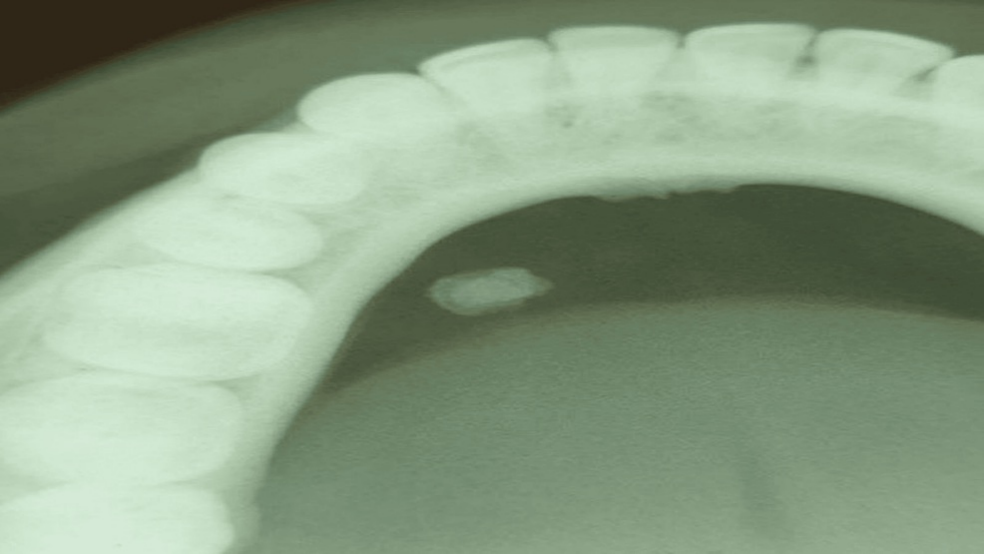
This is a mandibular cross-sectional occlusal view, which revealed a well-defined radio-opacity seen on the left side. Image Credit: Prasanna R. Sonar

**Figure 3:**
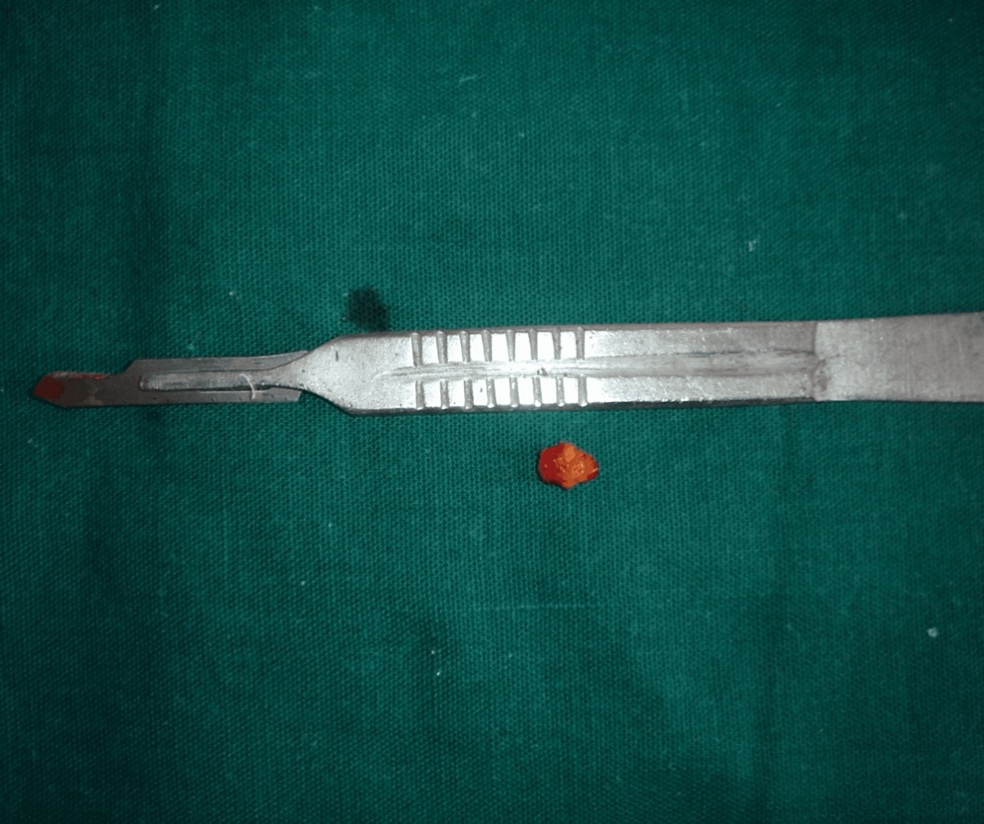
Submandibular gland sialolith of size 5 x 5 mm. Image Credit: Prasanna R. Sonar

**Figure 4:**
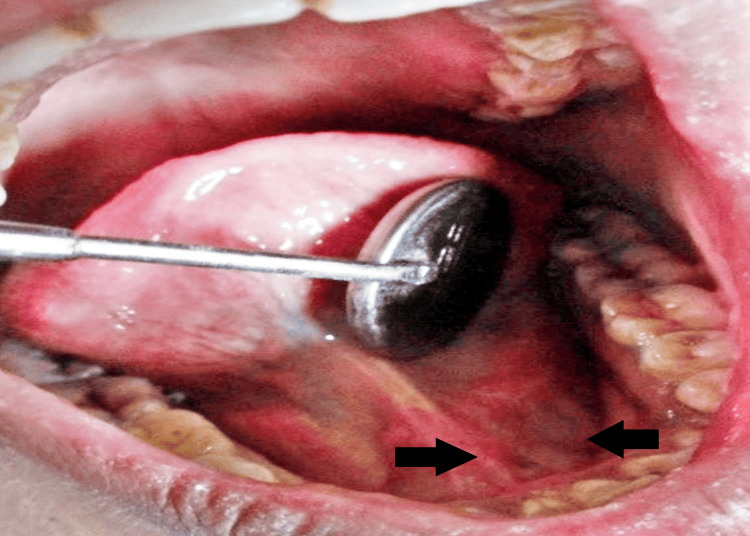
Recall visit intraoral photo. Image Credit: Prasanna R. Sonar

**Figure 5:**
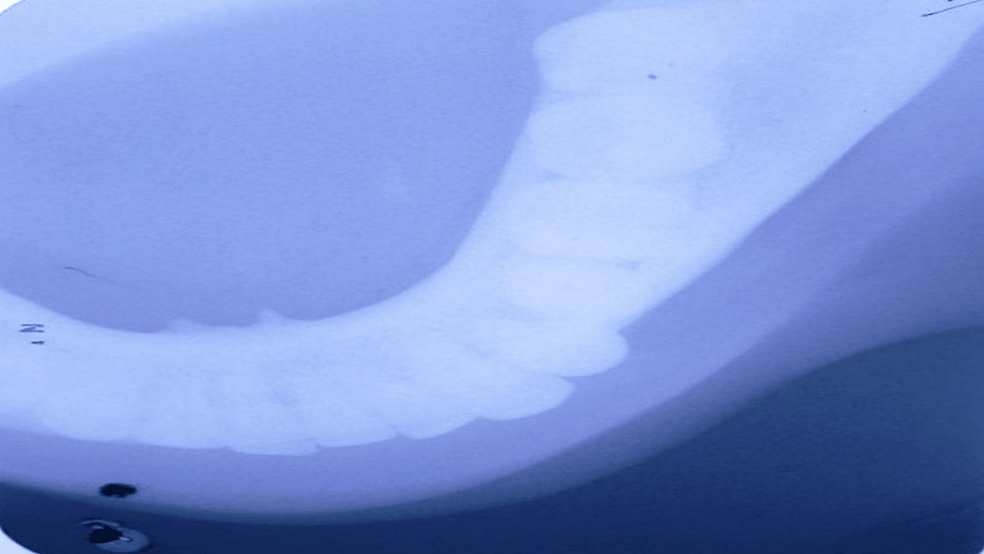
Recall visit cross-sectional mandibular occlusal radiograph showing no radio-opacity seen on the left side of the floor of the mouth. Image Credit: Prasanna R. Sonar

Upon intraoral inspection, a single diffuse swelling of about 1 x 1 cm approximately was found on the left side of the floor of the mouth. It has a smooth surface, a roughly oval form, and a similar color to the surrounding skin. Margins were diffuse on palpation as shown in Figure 1. There was discomfort with no pus discharge upon manipulation, and the consistency ranged from soft to hard. A well-defined radio-opacity that was radiographically indicative of sialolithiasis was seen on the left side of the mandible occlusal cross-sectional view as shown in Figure 2. Under local anesthesia, sialolith was removed following the opening of the duct. The wound was closed with sutures, and the patient was advised to practice tongue exercises and to maintain good oral hygiene. Figure 3 shows submandibular gland sialolith. The removed specimen was embedded in paraffin and preserved in 10% formalin. The tissues were immersed in paraffin and preserved in formalin and then sectioned into slices. Hematoxylin-eosin staining was done, and observations were made with a microscope. The specimen was primarily composed of highly mineralized laminated calculi. Other characteristics of the specimen were gland inflammation, parenchymal inflammation, duct ectasia, ductal epithelium hyperplasia, and squamous metaplasia. After histopathologic report, sialolithiasis in the left submandibular gland was given as the final diagnosis. The patient reported for follow-up after 15 days. Intraoral examination revealed no diffuse swelling present on the left side of the floor of the mouth as shown in Figure 4. Recall visit mandibular occlusal cross-sectional view revealed no radio-opacity seen on the left side of the floor of the intraoral cavity as shown in Figure 5.

## Discussion

The prevalent condition affecting the major and minor salivary glands is sialoliths. Both men and women are equally affected [3]. Since children's stones take longer to form and as age increases the amount of calcium and phosphate in the saliva at resting condition, they are not frequently seen in youngsters [4,5]. Seventy-five percent of cases of sialolithiasis are unilateral, 3% are bilateral, and 2% are atrophic [3].

The majority of these forms are caused by mineral salts building up around an organic nidus made up of salivary mucins, bacteria, and desquamated epithelial cells [4]. Sialoliths are assumed to form when mineral salts collect around an initial nidus made up of bacteria, desquamated epithelial cells, or salivary mucus; however, the exact etiology is yet unknown. They originate from the mineralization of debris that has gathered within the duct's lumen. This debris might consist of foreign substances, mucus plugs, exfoliated ductal epithelial cells, bacterial colonies, or other cellular debris. Calculus formation may be predisposed by conditions such as decreased crystalloid solubility, excessive alkalinity, elevated calcium content, dehydration, altered salivary pH linked to oropharyngeal infection, and trauma to the salivary duct or gland. Our instances' specific etiology is still a mystery. There are two stages of sialolith formation: layered periphery formation and central core building. First, mineral salts bonded by particular organic molecules precipitate, forming the core. Then, in the second phase, a few layers of inorganic and organic elements begin to accumulate around the central core. It is believed that a nidus of mucous and a swarm of foreign bodies or inflammatory cells are often the sites of parotid and submandibular stone formation [5,6].

Acute or chronic sialadenitis and shrinkage of the obstructed gland are the main effects of sialolithiasis. Long-term sialolith blockage can cause significant harm to the gland's acini, which could lead to a permanent reduction in salivary production or possibly its absence. Recurrent infections may result from this decreased or nonexistent salivary secretion, which may then cause the gland to atrophy, lose its secretory function, and eventually develop fibrosis [2].

The duct contains the majority of the stones in the submandibular gland. Out of all the small salivary glands, 10% are placed in the lower lip, 10% in the buccal mucosa, 47% in the upper lip, and 10% elsewhere. Only 10% of sialoliths are found in the lower lip. As seen in our case, the left submandibular gland is more prone than the right to generate a sialolith [7]. Due to characteristics like saliva's retrograde flow, the gland's enhanced production of calcium and mucus, the saliva's alkaline composition, and the duct's convoluted, tortuous, and lengthier course, submandibular glands are most commonly involved [4].

The patient typically has periods of remission after sporadic swelling and pain on the afflicted side during mastication [7]. Our patient likewise has these characteristics. Trismus, lymphadenopathy, pus discharge, and systemic infections are all linked to pain. No matter how big the stone is, Siddiqui claims that some patients can be asymptomatic [3]. Phleboliths, abscessed teeth, tonsillitis, calcified hemangiomas, cervical lymphadenopathy, and benign salivary gland tumors are among the differential diagnoses [4,8]. Appropriate imaging modalities, a clinical and physical examination, and a thorough history all contribute to the development of an accurate diagnosis and treatment strategy. The current diagnostic imaging methods used for salivary stone imaging are conventional radiography, magnetic resonance (MR) sialography, sialoendoscopy, ultrasound, computed tomography (CT), and sialography. The substantial radiation dose associated with CT imaging is the main disadvantage. When imaging salivary gland calculi, digital subtraction sialography and ultrasonography are the preferred techniques. Despite being a relatively ancient diagnostic technique, sialography remains the most effective diagnostic tool for imaging the complex architecture of the salivary gland duct system [1,9,10]. MR sialography is a noninvasive technique that doesn't involve cannulating the salivary duct opening, expose patients to ionizing radiation, or involve the administration of iodine contrast. In cases of acute salivary gland irritation, the examination may be performed. This is an alternative to digital subtraction sialography, particularly when salivary opening cannulation fails or there is acute sialadenitis. MR sialography makes it possible to precisely assess the morphology of the salivary ducts and see their tertiary branches [11]. Sialography is not recommended when calculus is present in the distal portion of the duct and the injected contrast medium prevents the calculus from being cleared by moving it toward the gland [3]. In these situations, scintigraphy is suggested. Since most submandibular lithiasis cases are radiopaque, not all of them can be detected by X-rays [7]. Since CT scans are noninvasive, they are the preferred method for diagnosing sialoliths [12]. Pachisia et al. stated that a significant drawback of ultrasonography is its inability to detect smaller calculi (>2 mm) [13]. When CT is unable to identify calculus, sialoendoscopy, a minimally invasive procedure that offers a clear picture of the ductal system, is recommended [8].

Sialendoscopy is one of the primary diagnostic instruments, enhancing the sensitivity of conventional imaging methods, and is advised for the diagnosis of sialadenosis, stenosis, foreign bodies, polyps, recurrent sialadenitis, and sialolithiasis. While other imaging techniques, even those that are less invasive, might only be helpful for diagnosis, sialendoscopy can be utilized in a single session for both diagnostic and therapeutic objectives. The goal of sialendoscopy therapy is to address the underlying cause of obstructive sialadenitis. Three phases make up the basic surgical procedure: identifying the papilla's position and introducing the sialendoscope, diagnosing, and initiating treatment. The papilla could be challenging to identify. As a result, a microscope or magnifying loupes may be useful. By massaging the gland with one hand until saliva leaks out, one can increase the papilla's visibility. Sialagogues such as lemon juice or ascorbic acid can help with this technique. Use lidocaine hydrochloride spray as a local anesthetic. The papilla's natural diameter is approximately 0.5 mm; thus, it must be dilated in order for the endoscope to pass through the duct. This can be done in a few different ways. By using progressively larger salivary probes to dilate the papilla, the "classic technique" is achieved. The "guided puncture technique" starts with the introduction of a smaller-diameter probe, which is progressively replaced with a titanium guide with the same diameter. This guide introduces a conical dilator to gradually stretch the papilla. After removing the dilatator, the endoscope is inserted through the working channel with the aid of the guide. After a proper ductal image is acquired, the guide is removed. When it's tough to locate the papilla, Nahlieli's "surgical" less cautious approach may be used. A meticulous incision is performed at the level of the oral pelvis, parallel to the duct's axis, on the medial aspect of the sublingual gland. After locating the duct, it must be opened by 1 mm in order to enter the endoscope without losing pressure while irrigation is taking place. This treatment is designated for cases where an atraumatic approach is not practical due to papillary hypertrophy, papillary stenosis, or extremely small ductal orifices [14-16].

The location and size of the sialolith determine the treatment plan for sialolithiasis. Noninvasive conservative care, which includes irrigation, sialagogue usage, gland massage after meals, and excessive water intake, is recommended when the stone is smaller in size. Numerous cutting-edge therapies have emerged, such as endoscopic laser lithotripsy, which breaks apart salivary stones by combining laser energy and sialendoscopy. To split the stone into tiny pieces, the laser is directed toward it through the endoscope. With this technique, the surrounding tissues sustain the least amount of injury while accurate stone fragmentation is possible. Treatment of bigger or difficult-to-reach stones has demonstrated positive outcomes. By introducing a tiny tube or stent into the salivary duct system, salivary duct stenting allows the stone to flow through or makes future treatments easier. Stenting can be done independently or in conjunction with sialendoscopy [13]. In situations of strictures or recurring sialolithiasis, it is especially helpful. High-energy shock waves are used in extracorporeal shock wave lithotripsy, a noninvasive therapy, to shatter large stones into tiny pieces. This makes it easier for the broken stones to flow through the duct system. For larger submandibular stones and stones in the parotid gland, this approach has demonstrated encouraging outcomes [3,13]. "Endoscopically assisted stone retrieval" in the sense of blind endoscopic stone retrieval using a Dormia basket, but not interventional sialendoscopy, is no longer advised, despite the fact that it is blinding and carries an elevated risk for perforation and ductal lesions. The size of the stones in the glands has an impact on the outcome of interventional sialendoscopy. Using a fiber-optic laser to fragment sialoliths is the most effective method. The laser is inserted into the sialendoscope, laser sialolithotripsy is carried out with direct visual supervision, and wire baskets are used to retrieve the broken stone. The recovery of sialoliths and their pieces following lithotripsy is advantageous [9].

An intraoral procedure performed under local anesthetic can remove most intraductal submandibular and parotid stones [17]. The preferred treatment modalities for large stones are invasive procedures like surgical removal while under general anesthesia, sialoendoscopy, CO2 laser-guided removal, endoscopic intracorporeal shock wave lithotripsy, or a combination of methods [13,18-22]. Based on the presenting problem, clinical characteristics, and CT results, we chose to remove sialolith under local anesthesia. Surgical methods can result in complications such as facial scarring, neurological impairment, salivary fistula, and Frey's syndrome [2]. Patients experience recurrence in 1-10% of cases [9]. During follow-up appointments, our patient has not reported any surgical problems or recurrences. Other treatment options for sialolithiasis include sialogogues with nonsteroidal anti-inflammatory drugs (NSAIDs) and direct stone removal from the duct via massage [1,23].

Patients should be informed that conservative treatment usually results in an excellent prognosis for sialolithiasis. They also need to be informed about typical initial symptoms like salivary gland swelling and intermittent pain in between or after meals. Although idiopathic sialolithiasis is the most common cause, obstructions like tumors or ductal stenosis can also result in stones. Patients should be informed on the importance of notifying their clinician if they experience repeated or worsening symptoms, since this may indicate the need for a more extensive diagnostic assessment or referral to an oral physician.

## Conclusions

Sialoliths should always be taken into account when a patient experiences face or submandibular pain, especially if it occurs around lunch. To define the exact position of the calcification and establish the clinical diagnosis, accurate imaging techniques and a thorough history are necessary. Even with the availability of more sophisticated and effective techniques, occlusal radiographs remain a valuable diagnostic tool for sialoliths. If better technologies are not available, the treatment of choice for larger stones is sialolithotomy combined with analgesics. Smaller stones can be treated conservatively.
